# Supplementary material for: HIVprotI: an integrated web based platform for prediction and design of HIV proteins inhibitors
Source: J Cheminform. 2018 Mar 9;10:12. doi: 10.1186/s13321-018-0266-y (PMC5845081; doi:10.1186/s13321-018-0266-y)
Supplement: Supplementary file 2 — Additional file 2. Source code of HIVProtI web server. [file 13321_2018_266_MOESM2_ESM.zip › HIVprotI_Source-code/hivprotI/weka-3-6-6/doc/weka/experiment/PropertyNode.html]

PropertyNode


---


|  |  |  |
| --- | --- | --- |
| **Overview** \| **Package** \| **Class** \| **Tree** \| **Deprecated** \| **Index** \| **Help** \| **Weka's home** | |  |
| **PREV CLASS**   **NEXT CLASS** | **FRAMES**    **NO FRAMES**     **All Classes** |  |
| SUMMARY: NESTED \| FIELD \| CONSTR \| METHOD | DETAIL: FIELD \| CONSTR \| METHOD |  |


---


## weka.experiment Class PropertyNode

```
java.lang.Object
  weka.experiment.PropertyNode
```

**All Implemented Interfaces:**: java.io.Serializable, RevisionHandler

---

``` public class PropertyNode extends java.lang.Object implements java.io.Serializable, RevisionHandler ```

Stores information on a property of an object: the class of the
object with the property; the property descriptor, and the current
value.

**Version:**
:   $Revision: 1.7 $

**Author:**
:   Len Trigg (trigg@cs.waikato.ac.nz)

**See Also:**: Serialized Form

---

| **Field Summary** | |
| --- | --- |
| `java.lang.Class` | `parentClass`             The class of the object with this property |
| `java.beans.PropertyDescriptor` | `property`             Other info about the property |
| `java.lang.Object` | `value`             The current property value |


**Constructor Summary**

`PropertyNode(java.lang.Object pValue)`
  
          Creates a mostly empty property.

`PropertyNode(java.lang.Object pValue,
java.beans.PropertyDescriptor prop,
java.lang.Class pClass)`
  
          Creates a fully specified property node.


| **Method Summary** | |
| --- | --- |
| `java.lang.String` | `getRevision()`             Returns the revision string. |
| `java.lang.String` | `toString()`             Returns a string description of this property. |

**Methods inherited from class java.lang.Object**

`equals, getClass, hashCode, notify, notifyAll, wait, wait, wait`

**Field Detail**

### value

```
public java.lang.Object value
```

:   The current property value

---


### parentClass

```
public java.lang.Class parentClass
```

:   The class of the object with this property

---


### property

```
public java.beans.PropertyDescriptor property
```

:   Other info about the property


**Constructor Detail**

### PropertyNode

```
public PropertyNode(java.lang.Object pValue)
```

:   Creates a mostly empty property.

    **Parameters:**: `pValue` - a property value.

---


### PropertyNode

```
public PropertyNode(java.lang.Object pValue,
                    java.beans.PropertyDescriptor prop,
                    java.lang.Class pClass)
```

:   Creates a fully specified property node.

    **Parameters:**: `pValue` - the current property value.: `prop` - the PropertyDescriptor.: `pClass` - the Class of the object with this property.


**Method Detail**

### toString

```
public java.lang.String toString()
```

:   Returns a string description of this property.

    :   **Overrides:**: `toString` in class `java.lang.Object`
    :   **Returns:**: a value of type 'String'

---


### getRevision

```
public java.lang.String getRevision()
```

:   Returns the revision string.

    :   **Specified by:**: `getRevision` in interface `RevisionHandler`
    :   **Returns:**: the revision


---


|  |  |  |
| --- | --- | --- |
| **Overview** \| **Package** \| **Class** \| **Tree** \| **Deprecated** \| **Index** \| **Help** \| **Weka's home** | |  |
| **PREV CLASS**   **NEXT CLASS** | **FRAMES**    **NO FRAMES**     **All Classes** |  |
| SUMMARY: NESTED \| FIELD \| CONSTR \| METHOD | DETAIL: FIELD \| CONSTR \| METHOD |  |


---
